# Isoperistaltic hand-sewn side-to-side bowel primary anastomosis: a safe approach after bowel resection in children with neutropenic enterocolitis

**DOI:** 10.1007/s00595-025-02998-z

**Published:** 2025-02-01

**Authors:** Mohammad Taher, Maged Elshafiey, Ahmed Refaat, Eman Nasr, Gehad Ahmed

**Affiliations:** 1 Department of Surgical Oncology, National Cancer Institute, Cairo University, El Kasr El Aini St., Fom El-Khaleeg Sq., Cairo, 11796 Egypt; 2 Department of Surgery, Children’s Cancer Hospital Egypt (CCHE, 57357), Cairo, Egypt; 3 Department of Radio Diagnosis, National Cancer Institute, Cairo University, Cairo, Egypt; 4 Department of General Surgery, Faculty of Medicine, Helwan University, Cairo, Egypt; 5 Children’s Cancer Hospital Egypt (CCHE, 57357), Cairo, Egypt

**Keywords:** Neutropenic enterocolitis, Typhlitis, Primary anastomosis, Side-to-side isoperistaltic anastomosis, Pediatric cancer, Stoma

## Abstract

**Background and aim:**

Whether to perform primary anastomosis (PA) or create a stoma after bowel resection has always been a dilemma in pediatric cancer patients with neutropenic enterocolitis (NEC). The risk of leakage after PA must be weighed against the risk of stoma complications. We evaluated the outcomes of managing NEC patients with either PA or stoma and the utility of the isoperistaltic hand-sewn side-to-side anastomosis (ISSA) technique in PA.

**Patients and methods:**

A retrospective study on all Children's Cancer Hospital Egypt patients with NEC who underwent surgical exploration at our hospital from 2008 to 2022.

**Results:**

Of 153 children, 80 (52.3%) underwent PA and 73 (47.7%) underwent stoma formation. Among the 80 PA patients, 68 (85%) underwent ISSA, 9 (11.2%) end-to-end anastomosis (EEA), and 3 (3.8%) end-to-side anastomosis (ESA). The perioperative complication rate was 38/73 (52.1%) in the stoma patients and 35/80 (43.8%) in the PA patients. Leakage occurred in 6/68 (8.8%) ISSA patients, 5/9 (55.6%) EEA patients, and 1/3 (33.3%) of ESA patients.

**Conclusions:**

In pediatric cancer patients with NEC, PA using ISSA after bowel resection is considered a better approach than any other anastomotic configuration.

## Introduction

Neutropenic enterocolitis (NEC) is a serious consequence of neutropenia [1–3]. Usually occurring as a consequence of aggressive chemotherapy in cases of acute leukemia [4]. It is characterized by ulceration and mural inflammation with intestinal necrosis [2, 3].

Surgical intervention should be considered in cases of uncontrolled gastrointestinal bleeding, perforation, and clinical deterioration of the patient despite conservative treatment [5]. A recent meta-analysis reported an overall mortality rate for NEC of 42.2% (95% confidence interval, 40.2–44.2%), indicating that surgery was not associated with a higher mortality risk than medical treatment [2]. Thus, early surgery before the development of organ failure might decrease mortality rates [6]. Since it mainly affects the right colon, surgery is typically right hemicolectomy with or without primary anastomosis [7].

The decision to perform PA or create a stoma presents a dilemma, especially in immunocompromised pediatric patients [8]. The risk of leakage after PA is a significant concern, particularly when working on a diseased bowel, as in patients with NEC [8].

In the present study, we evaluated the outcomes regarding the management of children with NEC who underwent bowel resection and followed by either PA or stoma construction and regarding the adoption of the isoperistaltic hand-sewn side-to-side anastomosis (ISSA) technique for PA in these patients.

## Patients and methods

### Study design

A retrospective study on all Children Cancer Hospital Egypt (CCHE) patients with NEC who underwent surgical exploration from January 2008 to December 2022.

### Methodology

The Institutional Review Board (IRB) at the Children’s Cancer Hospital Egypt (CCHE, 57,357) approved the study, and the informed consent of patients was waived. IRB approval serial number: 65/2023.

Patients’ data were collected from medical records, and the following items were obtained and analyzed:

- *Preoperative data:* inotropic agents used, blood culture, degree of neutropenia, radiological localization, mural thickness, complications as perforation, ischemia or obstruction.
- *Operative data:* operative findings, procedure, technique of anastomosis, causes of stoma formation (no PA) if performed.
- *Postoperative data:* postoperative complications such as a leak, wound infection, dehiscence, or reoperation.

The neutropenia grading scale was adapted from the Common Terminology Criteria for Adverse Events (CTCAE) Version 5.0 and categorized as follows: ≥ 4000/mm^3^ (G-0), < 4000–1500 (G-1), < 1500–1000 (G-2), < 1000–500 (G-3), and < 500 (G-4) [9].

### ISSA surgical technique

The diseased bowel segment was resected using the gastrointestinal anastomosis (GIA) linear stapler. A second layer of Vicryl 3–0 was placed over both staple lines continuously. Both ends were then set aside opposite each other to adopt the isoperistaltic configuration. Antimesenteric transverse incisions (about 6 cm) were made at both ends, leaving no more than 3 cm of the edge of each end. Side-to-side anastomosis was performed in a hand-sewn fashion using Vicryl 3–0 in two layers: a continuous full-thickness layer and then an interrupted seromuscular layer. The mesenteric defect, which is usually small in size with this technique, was closed.

### Statistical analyses

Numeric data were presented as the mean ± standard deviation or median and interquartile range. Categorical data were presented as percentages and absolute numbers. The clinical characteristics of the stoma and primary anastomosis groups were compared. The factors associated with postoperative complications were also analyzed. The significance of these distributions was assessed by the chi-square or Fisher's exact test for categorical values and Wilcoxon’s test for numeric values. The failure rate was calculated as the percentage of anastomotic leak. A univariate regression analysis assessed the link between each variable and the probability of a leak. Factors that showed significant or marginal significance in the unadjusted analysis were considered in the adjusted analysis using multivariate logistic regression. For significant predictors, likelihood odds ratios with corresponding 95% confidence intervals (CIs) were estimated. P values < 0.05 were considered significant. Statistical analyses were performed using SPSS Statistics software program, version 22 for Windows (SPSS, Inc., IBM Corporation, NY, USA).

## Results

Our study included 153 children with neutropenic enterocolitis who underwent surgical exploration after initiation of conservative measures. Most cases had hematological malignancies (116, 75.8%; leukemias in particular) and high-grade neutropenia (150, 98%; Grade 4, count < 500/mm^3^). The indications for surgical interference were grouped into two main categories: those whose general condition deteriorated despite conservative treatment and those who developed complications necessitating surgery, such as perforation, obstruction, severe perianal sepsis, and bowel ischemia or gangrene.

We divided the patients into two groups: those who underwent PA without stoma coverage (n = 80, 52.3%) and those who underwent a stoma with or without distal anastomosis (n = 73, 47.7%). On comparing the characteristics between the stoma and PA groups, we found that most were comparable. The stoma patients, however, were significantly more frequently associated with diffuse and multifocal disease, inotropes used in the preoperative setting, and decreased reoperation rates (p-values of < 0.001, 0.03, and 0.001, respectively; Table 1).

**Table 1:** Patients’ characteristics

Characteristic		Total	Surgical intervention		p-value
			Stoma (n = 73)	Primary anastomosis without stoma (n = 80)	
Age at surgery (years)	Mean ± SD Median (Min–Max)	7.6 ± 4.7 6.5 (0.1–21.6)	7.5 ± 4.6 6.1 (0.5–21.6)	7.7 ± 4.9 6.6 (0.1–18.4)	**0.7**
Gender	Female Male	52 (34%) 101 (66%)	23 (31.5%) 50 (68.5%)	29 (36.3%) 51 (63.7%)	**0.5**
Primary Diagnosis	Hematological malignancy Non-hematological malignancy	116 (75.8%) 37 (24.2%)	55 (75.3%) 18 (24.7%)	61 (76.3%) 19 (23.7%)	**0.9**
Grade of Neutropenia (/mm^3^)^*^	< 1500–1000 (G-2) < 500 (G-4)	3 (2%) 150 (98%)	0 73 (100%)	3 (3.8%) 77 (96.2%)	**0.09**
Presence of other septic focus/foci	No other septic focus Other septic focus / foci	73 (47.7%) 80 (52.3%)	33 (45.2%) 40 (54.8%)	40 (50%) 40 (50%)	**0.6**
Blood Culture	Negative Positive	129 (84.3%) 24 (15.7%)	65 (89%) 8 (11%)	64 (80%) 16 (20%)	**0.1**
Disease extent and focality	Diffuse Localized unifocal Localized multifocal	86 (56.2%) 37 (24.2%) 30 (19.6%)	59 (80.8%) 5 (6.9%) 9 (12.3%)	27 (33.8%) 32 (40%) 21 (26.2%)	** < 0.001**
Radiological Mural thickness (mm)	Mean ± SD Median (Min–Max)	15 ± 6 15 (4–40)	15.2 ± 5.7 15 (7–40)	15.7 ± 6.1 15.5 (7–34)	**0.6**
Patient was on Inotropes preoperatively	No Yes	85 (55.6%) 68 (44.4%)	34 (46.6%) 39 (53.4%)	51 (63.7%) 29 (36.3%)	**0.03**
Indication for Surgical intervention	Deterioration of general condition Complications necessitating surgery	78 (51%) 75 (49%)	33 (45.2%) 40 (54.8%)	45 (56.2%) 35 (43.8%)	**0.2**
Perioperative Complications	No Yes	80 (52.3%) 73 (47.7%)	35 (47.9%) 38 (52.1%)	45 (56.2%) 35 (43.8%)	**0.4**
Surgical management of complications (i.e. reoperation)	No Yes (of complicated cases)	48 (65.8%) 25 (34.2%)	32 (84.2%) 6 (15.8%)	16 (45.7%) 19 (54.3%)	**0.001**
Postoperative 30-day mortality	No Yes	84 (54.9%) 69 (45.1%)	39 (53.4%) 34 (46.6%)	45 (56.3%) 35 (43.7%)	**0.7**

The causes that necessitated to construct a stoma were as follows: diffuse bowel disease in 38 (52%) patients, anorectal disease in 13 (17.8%), resection at multiple levels in 4 (5.5%), and unstable intraoperative condition that necessitates to shorten the procedure time in 18 (24.7%) patients. We adopted 3 techniques for anastomotic reconstruction, and among the 80 patients who underwent PA construction, 68 (85%) had ISSA, 9 (11.2%) had end-to-end anastomosis (EEA), and 3 (3.8%) had end-to-side anastomosis (ESA). The technique tended to be chosen according to surgeon's preference with no solid bases otherwise at that time.

The perioperative complication rate was 47.7% (73/153), including 52.1% (38/73) in the stoma group and 43.8% (35/80) in the PA group. Details of complications are shown in Table 2. A total of 69 (45%) cases experienced 30-day postoperative mortality, and the 30-day mortality rates for the stoma and PA groups were 46.6% and 43.75%, respectively. No statistically significant differences were found between both groups regarding perioperative morbidity and mortality, as shown in Table 1.

**Table 2:** Details of perioperative complications

Complication	Primary anastomosis-group (n = 80)	Stoma –group (n = 73)	Total (n = 153)
Leakage	12 (15%)	NA	12 (7.8%)
Intestinal obstruction	2 (2.6%)	0	2 (1.3%)
Intraabdominal abscess	3 (3.8%)	0	3 (2%)
Burst abdomen	1 (1.3%)	2 (2.7%)	3 (2%)
Wound infection	9 (11.3%)	8 (11%)	17 (11.1%)
Prolapsed stoma	NA	1 (1.4%)	1 (0.7%)
Skin maceration	0	7 (9.6%)	7 (4.6%)
Electrolyte disturbance	1 (1.3%)	8 (11%)	9 (5.9%)
Bleeding from stoma sites	NA	7 (9.6%)	7 (4.6%)
Sepsis	4 (5%)	4 (5.4%)	8 (5.2%)
Chest infection	3 (3.8%)	1 (1.4%)	4 (2.6%)
Total number	35 (43.7%)	38 (52.1%)	73 (47.7%)

NA Not Applicable

Leakage occurred in 12 of the 80 PA patients (15%), including 6 of 68 (8.8%) ISSA cases, 5 of 9 (55.6%) EEA cases, and 1 of 3 (33.3%) ESA cases. The difference in the leakage rate according to the type of anastomosis was statistically significant, with a p-value of 0.001. Ten patients had a major (> 500 cc/day) leak (4 ISSA, 5 EEA, and 1 SEA), and 2 had a minor (≤ 500 cc/day) leak (all ISSA).

Positive blood culture was another significant negative prognostic factor for leakage occurrence, with a p-value of 0.04. In the multivariate analysis, the anastomotic technique was the only significant factor associated with leakage with a p-value of 0.004, a hazard ratio (HR) of 8.66 (95% CI 2–36.7) for techniques other than ISSA. Table 3 presents these factors.

**Table 3:** Univariate and multivariate analyses of factors associated with leakage in patients underwent primary anastomosis (n=80)

Univariate analysis				
Characteristic		Leakage		P-value
		No	Yes	
Disease extent	Generalized Localized	21 (77.8%) 47 (88.7%)	6 (22.2%) 6 (11.3%)	0.197
Primary diagnosis	Hematological malignancy Non-hematological malignancy	51 (83.6%) 17 (89.5%)	10 (16.4%) 2 (10.5%)	0.532
Grade of neutropenia	< 1500–1000 < 500	2 (66.7%) 66 (85.7%)	1 (33.3%) 11 (14.3%)	0.365
Anastomotic technique	SSA (side to side) ESA (end to side) EEA (end to end)	62 (91.2%) 2 (66.7%) 4 (44.4%)	6 (8.8%) 1 (33.3%) 5 (55.6%)	**0.001**
Other septic focus/foci	No other septic focus other septic focus / foci	33 (82.5%) 35 (87.5%)	7 (17.5%) 5 (12.5%)	0.531
Indication of surgery	Deterioration of general condition Complications necessitating surgery	41 (91.1%) 27 (77.1%)	4 (8.9%) 8 (22.9%)	0.083
Blood culture	Negative Positive	57 (89.1%) 11 (68.8%)	7 (10.9%) 5 (31.3%)	**0.042**
Patient on inotropes preoperatively	No Yes	43 (84.3%) 25 (86.2%)	8 (15.7%) 4 (13.8%)	0.82

Multivariate analysis								
Characteristic	B	S.E	Wald	df	Sig	Exp(B)	95% CI for EXP(B)	
							Lower	Upper
Anastomotic technique (ISSA vs others), ref: ISSA	2.155	0.738	8.516	1	**0.004**	8.627	2.029	36.683
Blood culture, ref: positive	0.931	0.749	1.546	1	0.214	2.538	0.585	11.014

Bold indicates statistically significant p values

The two patients with minor leaks were managed conservatively, while the 10 patients who developed major leaks underwent reoperation with stoma constructed in three of them (2 EEA, and 1 SEA). These three patients were found to have gross massive fecal peritonitis with a diffusely diseased bowel wall and thus died within two weeks due to peritonitis and subsequent septicemia. The other seven patients with major leakage had mild if any localized peritonitis without any gross contamination, and refashioning of the anastomosis was done, thus resulting in a smooth recovery.

## Discussion

The dearth of high-quality research makes it difficult to standardize proposals on the approach for NEC. Treatment recommendations are based on descriptive studies or clinical experience [1]. Surgical management has been considered safe with a better understanding of the disease, and it should be encouraged in certain situations after initiating conservative measures [1, 2, 5, 6].

Previous studies have thoroughly discussed and compared the options of operative vs. nonoperative management of NEC children; however, the research is deficient in terms of discussing the best and safest surgical options. Most articles discussing the feasibility of primary anastomosis and the safest techniques when choosing this option present isolated case reports or studies involving limited numbers of patients. For example, a systematic review and meta-analysis study by Saillard et al. included 20 studies on 385 patients over a period of 33 years [2]. We therefore referenced and compared our results to similar diseases in terms of the pathogenesis, pattern of affection, and surgical management of its complications. Based on this, we deemed necrotizing enterocolitis and Crohn's diseases to be our reference diseases.

While NEC is a panintestinal disease, affection is mostly ominous in the ileocecal region. Therefore, if surgery is indicated, right hemicolectomy and a defunctioning ileostomy are routinely adopted as the standard. The inclination towards stoma creation in neutropenic patients is based on the notion that a primary intestinal anastomosis is doomed to fail when sepsis or peritoneal infection is present alongside a microscopically ischemic bowel [10, 11]. Other disputes against PA are the surgeons' inability to judge the disease extent, potentially resulting in a greater extent of resection than required [10, 12].

This concept has been challenged, particularly in neutropenic patients, because of the stoma-related complication rate and the burden it adds to the child, as documented in many series [13–15]. Consequently, PA has been prioritized whenever feasible in recent years [10, 16–18]. In the present study, 52.3% underwent PA without a covering stoma, while 47.7% of patients had a stoma. In a study by Sweed et al., 9 patients had stomas, and 22 had PA [16]. Guelfand et al. also stated that PA was performed in 60 newborn patients with necrotizing enterocolitis (85%), whereas 10 (15%) had a stoma [10]. However, other studies had a high stoma construction rate, similar to the present study, like Ramaswamy et al., who stated that 6 patients underwent resection with PA, while 16 underwent stoma formation [19].

Our complication rate was 47.7% overall, 52.1% in the stoma group, and 43.8% in the PA group. Other studies, however, documented lower complication rates. Ramaswamy et al. stated that they had 6 (27.3%) complications (2 [33.33%] in the anastomosis group and 4 [25%] in the stoma group) [19]. Guelfand et al. reported a stoma-related complication rate of 68% and an anastomosis-related complication rate of 16.7% [10]. Similarly, Sweed et al.’s short-term outcome results showed that 3 of 9 infants (33%) in the stoma group had surgery-related complications, while none of the PA infants had surgery-related complications [16]. In the present study, leakage developed in 12 of 80 PA patients (15%). Other studies documented lower rates of leakage, including 42/477 (8.8%), 1/60 (1.7%), and 0% in studies by Sánchez-Guillén et al., Guelfand et al., and Sweed et al., respectively [10, 16, 20].

A sideways anastomosis is believed to provide superior blood flow compared to an end anastomosis, along with a larger diameter, which leads to reduced intraluminal pressure and decreased proximal ischemia [21]. Although different studies favor the antiperistaltic technique since it has more functionality due to the pseudovalvular mechanism which replaces the missing ileocecal valve [22, 23], the isoperistaltic technique is favored with a diseased bowel, such as in cases of NEC, to allow a wider lumen, functional peristalsis, easier passage of contents without pressure difference that may cause stenosis or obstruction, and avoidance of excessive mobilization of the bowel with more ischemia [24–26]. Our findings corroborated this point, revealing the leakage rate to be lowest among patients who underwent ISSA.

The stoma creation and complication rates in our study are marginally higher than in other studies. This discrepancy may be due to variations in perioperative patient conditions, selection criteria, and the surgical indication thresholds utilized across studies.

Of the 73 perioperative complications in our study, 25 underwent at least one operation that required subsequent management, making our reoperation rate 34.2%. Among stoma patients, this rate was 6/38 (15.8%), and among PA patients, it was 19/35 (54.3%). Thus, we had a much higher reoperation rate among PA patients than stoma patients, mostly due to the leakage complication that is not seen in stoma patients. In contrast, Sweed et al. found the reoperation rate to be 11 (35.5%), 6 (66.7%), and 5 (22.7%) overall and in the stoma and PA groups, respectively, with the rate being highest among stoma patients [16].

Several limitations associated with the present study warrant mention, such as its retrospective nature and the lack of randomization for the selected anastomotic technique. In addition, patients in the stoma group typically exhibit poorer general conditions than those in the PA group, rendering the comparison between the two groups invalid. We propose performing a prospective study to establish valid conclusions concerning the safety of PA and the optimal anastomotic technique in children with NEC.

## Conclusion

In pediatric cancer patients with neutropenic enterocolitis, primary anastomosis via the ISSA technique after bowel resection is considered a better approach than any other anastomotic configuration, with advantages of better vascularity and a lower rate of leakage. The safety and feasibility of PA, along with the establishment of a standard safe anastomotic technique for these patients, require further prospective studies to validate such critical concerns.

## Data Availability

The datasets used/analyzed during this study are available from the corresponding author on request.

## Abbreviations

CI: Confidence interval
CTCAE: Common Terminology Criteria for Adverse Events
EEA: End-to-end anastomosis
ESA: End-to-side anastomosis
HR: Hazard ratio
ISSA: Isoperistaltic hand-sewn side-to-side anastomosis
NEC: Neutropenic enterocolitis
PA: Primary anastomosis

## References

[1] Babakhanlou R, Ravandi-Kashani F, Kontoyiannis DP. Neutropenic enterocolitis: an uncommon, but fearsome complication of leukemia. J Hematol. 2023;12:59. 37187499 10.14740/jh1105 PMC10181327

[2] Saillard C, Zafrani L, Darmon M, Bisbal M, Chow-Chine L, Sannini A, et al. The prognostic impact of abdominal surgery in cancer patients with neutropenic enterocolitis: a systematic review and meta-analysis, on behalf the groupe de recherche en réanimation respiratoire du patient d’onco-hématologie (GRRR-OH). Ann Intensive Care. 2018. 10.1186/s13613-018-0394-6. 29675758 10.1186/s13613-018-0394-6 PMC5908777

[3] Sachak T, Arnold MA, Naini BV, Graham RP, Shah SS, Cruise M, et al. Neutropenic enterocolitis. Am J Surg Pathol. 2015;39:1635–42. 26414225 10.1097/PAS.0000000000000517

[4] Gorschlüter M, Mey U, Strehl J, Ziske C, Schepke M, Schmidt-Wolf IGH, et al. Neutropenic enterocolitis in adults: systematic analysis of evidence quality. Eur J Haematol. 2005;75:1–13. 15946304 10.1111/j.1600-0609.2005.00442.x

[5] Altinel E, Yarali N, Isik P, Bay A, Kara A, Tunc B. Typhlitis in acute childhood leukemia. Med Princ Pract. 2011;21:36–9. 22024548 10.1159/000331587

[6] Vergara-Fernández O, Trejo-Avila M, Solórzano-Vicuña D, Santes O, Salgado-Nesme N. Factors associated with emergent colectomy in patients with neutropenic enterocolitis. Langenbeck’s Arch Surg. 2019;404:327–34. 30953135 10.1007/s00423-019-01781-2

[7] Urbach DR, Rotstein OD. Typhlitis. Can J Surg. 1999;42:415–9. 10593241 PMC3795130

[8] Rokhsar S, Harrison EA, Shaul DB, Phillips JD. Intestinal stoma complications in immunocompromised children. J Pediatr Surg. 1999;34:1757–61. 10626848 10.1016/s0022-3468(99)90306-8

[9] Cancer Institute N. Common terminology criteria for adverse events (CTCAE) common terminology criteria for adverse events (CTCAE) v5.0. 2017; https://www.meddra.org/. Accessed 29 Feb 2024.

[10] Guelfand M, Santos M, Olivos M, Ovalle A. Primary anastomosis in necrotizing enterocolitis: the first option to consider. Pediatr Surg Int. 2012;28:673–6. 22526554 10.1007/s00383-012-3092-8

[11] Mullassery D, Bader A, Battersby AJ, Mohammad Z, Jones ELL, Parmar C, et al. Diagnosis, incidence, and outcomes of suspected typhlitis in oncology patients-experience in a tertiary pediatric surgical center in the United Kingdom. J Pediatr Surg. 2009;44:381–5. 19231539 10.1016/j.jpedsurg.2008.10.094

[12] Kiesewetter WB, Taghizadeh F, Bower RJ. Necrotizing enterocolitis: Is there a place for resection and primary anastomosis? J Pediatr Surg. 1979;14:360–3. 480100 10.1016/s0022-3468(79)80500-x

[13] Bælum JK, Rasmussen L, Qvist N, Ellebæk MB. Enterostomy complications in necrotizing enterocolitis (NEC) surgery, a retrospective chart review at Odense university hospital. BMC Pediatr. 2019;19:1–5. 10.1186/s12887-019-1488-5. 30979367 10.1186/s12887-019-1488-5 PMC6461813

[14] Mutanen A, Pierro A, Zani A. Perioperative complications following surgery for necrotizing enterocolitis. Eur J Pediatr Surg. 2018;28:148–51. 10.1055/s-0038-1636943. 29534255 10.1055/s-0038-1636943

[15] Massenga A, Chibwae A, Nuri AA, Bugimbi M, Munisi YK, Mfinanga R, et al. Indications for and complications of intestinal stomas in the children and adults at a tertiary care hospital in a resource-limited setting: a Tanzanian experience. BMC Gastroenterol. 2019;19:1–10. 10.1186/s12876-019-1070-5. 31462228 10.1186/s12876-019-1070-5 PMC6714288

[16] Sweed Y. The outcome of primary anastomosis and the long term follow up of preterm infants undergoing surgery for necrotizing enterocolitis. Acad J Pediatr Neonatol. 2020. 10.19080/AJPN.2020.09.555821.

[17] Singh M, Owen A, Gull S, Morabito A, Bianchi A. Surgery for intestinal perforation in preterm neonates: anastomosis vs stoma. J Pediatr Surg. 2006;41:725–9. 16567184 10.1016/j.jpedsurg.2005.12.017

[18] Haricharan RN, Gallimore JP, Nasr A. Primary anastomosis or ostomy in necrotizing enterocolitis? Pediatr Surg Int. 2017;33:1139–45. 10.1007/s00383-017-4126-z. 28770340 10.1007/s00383-017-4126-z

[19] Ramaswamy R, Hegab SM, Mugheri A, Mukattash G. Surgical treatment of necrotizing enterocolitis: Single-centre experience from Saudi Arabia. Ann Pediatr Surg. 2016;12:43–6.

[20] Sánchez-Guillén L, Frasson M, García-Granero PG, Flor-Lorente B, Álvarez-Sarrado E, et al. Risk factors for leak, complications and mortality after ileocolic anastomosis: comparison of two anastomotic techniques. Ann R Coll Surg Engl. 2019;101:571–8. 31672036 10.1308/rcsann.2019.0098 PMC6818057

[21] Simillis C, Purkayastha S, Yamamoto T, Strong SA, Darzi AW, Tekkis PP. A meta-analysis comparing conventional end-to-end anastomosis vs other anastomotic configurations after resection in Crohn’s disease. Dis Colon Rectum. 2007;50:1674–87. 17682822 10.1007/s10350-007-9011-8

[22] Ibáñez N, Abrisqueta J, Luján J, Hernández Q, Rufete MD, Parrilla P. Isoperistaltic versus antiperistaltic ileocolic anastomosis. Does it really matter? Results from a randomised clinical trial (ISOVANTI). Surg Endosc. 2019;33:2850–7. 10.1007/s00464-018-6580-7. 30426254 10.1007/s00464-018-6580-7

[23] Ibañez N, Abrisqueta J, Luján J, Hernández Q, Parrilla P. Isoperistaltic versus antiperistaltic side-to-side anastomosis after right laparoscopic hemicolectomy for cancer (ISOVANTI) trial: study protocol for a randomised clinical trial. Int J Colorectal Dis. 2017;32:1349–56. 28634703 10.1007/s00384-017-2840-6

[24] Chen W, Zhou J, Chen M, Jiang C, Qian Q, Ding Z. Isoperistaltic side-to-side anastomosis for the surgical treatment of Crohn disease. Ann Surg Treat Res. 2022;103:53–61. 35919111 10.4174/astr.2022.103.1.53 PMC9300438

[25] Michelassi F, Mege D, Rubin M, Hurst RD. Long-term Results of the side-to-side isoperistaltic strictureplasty in Crohn disease: 25-year follow-up and outcomes. Ann Surg. 2020;272:130–7. 30720502 10.1097/SLA.0000000000003221

[26] Maddock WG. Side-to-side ileotransverse colostomy, the anastomosis of choice. Surg Clin North Am. 1961;41:91–3. 10.1016/S0039-6109(16)36289-2. 13765047 10.1016/s0039-6109(16)36289-2

